# Is Reticular Macular Disease a Choriocapillaris Perfusion Problem?

**Published:** 2012

**Authors:** Miguel A. Martillo, Marcela Marsiglia, Michele D. Lee, Nicole Pumariega, Srilaxmi Bearelly, R. Theodore Smith

**Affiliations:** 1 Department of Ophthalmology, New York University, New York, USA; 2 Department of Ophthalmology, Harkness Eye Institute, Columbia University, New York, USA; 3 LuEsther T. Mertz Retinal Research Center and Manhattan Eye, Ear and Throat Hospital, New York USA

**Keywords:** Reticular macular disease, Choriocapillaris blood flow, Inflammation, Systemic diseases

## Abstract

The etiology of reticular macular disease (RMD), a sub-phenotype of age-related macular degeneration (AMD), is controversial and has not been clarified. RMD is suspected to be a multifactorial, complex disease with genetic, environmental, and systemic factors playing an important role in its origin. Findings from combinations of different imaging modalities suggest that the pattern that characterizes this condition is associated with an alteration of the choriocapillaris blood flow. If the choroid is indeed affected in RMD, the possible linkage with inflammatory or other systemic diseases could be better supported.

## INTRODUCTION

Age-related macular degeneration (AMD) is a degenerative disease of the macula that results in loss of central vision and is the leading cause of adult blindness in industrialized countries [1]. Late-stage AMD may occur in two different forms: neovascular AMD, also known as choroidal neovascularization (CNV) or “wet AMD,” and geographic atrophy (GA), also known as late-stage “dry” AMD. The Eye Disease Prevalence Research Group has studied AMD prevalence in people over 40 years old. According to this group, the prevalence for both forms of late-stage AMD is 1.47% in the United States, affecting 1.75 million people. They estimate a projected affected population of 3 million people by 2020 [2]. While there are treatments available for wet AMD, there has been no proven effective treatment for GA. The only treatment option currently available for early AMD is antioxidant vitamin therapy, which shows a trend to benefit for prevention of GA per the Age-Related Eye Disease Study [3]. Thus, there is a need to better characterize the disease and develop treatment options for high-risk individuals.

Drusen, which are localized deposits of extracellular material concentrated in the macula under the retinal pigment epithelium (RPE), have an established link with AMD. Recently, reticular pseudodrusen (RPD) have been described and associated with progression to late-stage AMD, especially CNV [4, 5]. RPD were first described in 1990 as a peculiar yellowish pattern in the macula of patients with AMD. Because their visibility was enhanced by the use of blue light, they were described as “les pseudodrusen bleus” [6]. Since then, numerous imaging modalities have been used to investigate this pattern.

Using color fundus photographs, Klein et al. identified an ill-defined network of broad, interlacing yellowish lesions occurring mainly in the outer macula and, based on their characteristic appearance, made these a separate entity (“reticular drusen”) in the Wisconsin Age-Related Maculopathy Grading System [7]. These were considered a type of drusen until Arnold et al. published a study on RPD that included a histopathological analysis of these lesions, which demonstrated a significant loss of the middle choroidal layer of small vessels and increased spacing between the large choroidal veins. This led to the hypothesis that fibrotic replacement of the choroidal stroma and loss of vascularity were responsible for the development of RPD, and RPD were proposed as a marker for choroidal ischemia [8].

**Figure 1:**
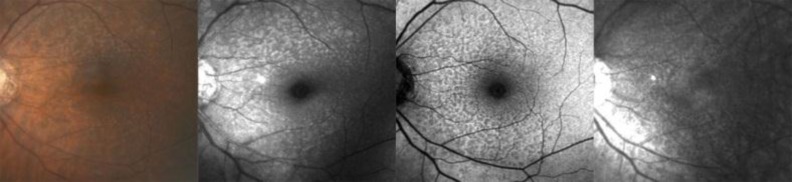
Classic presentation of RMD in fundus photography identified as yellow or light interlacing networks ranging from 125 to 250 microns in width in color (first image) and red free (second image) images [8]. Reticular AF pattern, hypoautofluorescent lesions against a background of elevated AF (third image) [11, 14]. Reticular IR pattern, hyporeflectant lesions against a background of hyperreflectance (fourth image) [11

Advances in imaging technology, particularly scanning laser ophthalmoscopy (SLO), have led to improved imaging and visualization of RPD. Unlike soft drusen, these lesions were observed to be hypofluorescent in the mid-to-late phases of indocyanine green angiography (ICGA) [9], with well-defined reticular patterns visualized with autofluorescence (AF) imaging [10]. Smith et al. demonstrated the characteristic appearances of reticular macular disease (RMD) using various imaging modalities, including SLO imaging (**see **Figure 1). They demonstrated spatial correspondence of individual lesions between imaging modalities, with the total areas of the macula involved either largely overlapping or falling within one another, suggesting that a single disease is responsible for each of these presentations [11]. The term “reticular macular disease (RMD)” includes RPD identified in color fundus photography or a reticular pattern seen in SLO, or both [11].

Klein and colleagues reported an overall RPD prevalence of 0.7% in the general population and a 15-year cumulative incidence of 3.0% in a population aged 43 to 86 years at baseline [12]. Furthermore, they reported a high 15-year cumulative incidence of late AMD (43% and 46% in right and left eyes, respectively) and a 54% poorer survival rate in those with RPD at baseline. A large epidemiologic study reported female gender as a major risk factor for RPD (OR = 2.59) and a possible association between RPD and systemic diseases [12]. In addition, the prevalence of RMD in GA has been reported to be as high as 62% [13], and a large prospective study demonstrated that, while RMD occurs in only 7-8% of patients with AMD overall [14], RMD was present in around 30% of patients with CNV, with double the risk of progression to CNV in the fellow eye when the CNV was unilateral [4].

Different genetic loci have been identified and associated with an increased risk for AMD progression. Through genome-wide scanning and the candidate gene approach, complement factor H (*CFH* [Y402H, rs1061170]) on the chromosome 1q32 locus is known to be a risk haplotype-tagging variant for AMD [15]. Another genetic locus identified was the age-related maculopathy susceptibility gene (*ARMS2 *[A69S, rs10490924]) on chromosome 10q26 [16-18]. Based on preliminary genetic results, RMD appears to be highly associated with the *ARMS2* risk allele, whereas the *CFH* risk variant is not associated with RMD [19].

From these recent discoveries, we suspect that RMD is a multifactorial, complex disease, with genetic, environmental, and systemic (inflammatory or vascular) factors playing an important role in its natural course. 

## HYPOTHESIS

We postulate that the reticular pattern identified as RMD in different imaging modalities is related to an alteration in choriocapillaris blood flow. Indeed, many multimodal imaging studies have suggested this etiology [11, 20, 21]. This hypothesis is conceived from the experience of our research group with patients that present with RMD and our review of other groups’ studies.

## DISCUSSION

We support our hypothesis by relying on imaging findings reported in studies that enabled detailed characterization of retinal layers and localized a possible origin of RMD. The use of optical coherence tomography (OCT) has allowed for better visualization of the choroid and retinal layers and increased our understanding of the important role of the choroid in RMD. Using en face OCT and precise image registration with AF, infrared (IR), and red free (RF) images, Sohrab et al. demonstrated that the arrangement and pattern of RPD are closely related to the choroidal stroma and the choroidal vasculature; reticular lesions followed the pattern of the underlying choroidal stroma on en face OCT and closely abutted larger choroidal vessels [20]. In another study using ICGA in addition to OCT, reticular areas appeared as hypofluorescent lesions adjacent to the large choroidal vessels [21]. Arnold et al. highlighted that the pattern seen in fluorescein angiography (FA) and ICGA is different in patients that have RMD and in those who have traditional drusen deposits. RMD is seen as distinctive groupings of hypofluorescent dots present in the mid-to-late phases of ICGA [9].

Rudolf et al. histologically described certain drusen-like lesions in AMD as “subretinal drusenoid deposits” [22]. Later studies [23-26] used OCT to describe discrete collections of interconnected hyperreflective deposit material above the RPE (unlike typical drusen, which are under the RPE), which were consistent with the original description of pseudodrusen, referring to them as “subretinal drusenoid deposits.” However, Querques et al. described corresponding areas of iso/hyperfluorescence on ICGA and IR adjacent to RPD lesions seen on OCT as subretinal deposits with inner segment/outer segment (IS/OS) disruption [21]. Similarly, Sohrab et al. used en face OCT to reveal subretinal hyperreflective deposits adjacent to RPD lesions on IR and overlying large choroidal vessels [20]. Given the close proximity to areas of hyperfluorescence, subretinal deposits may represent secondary mechanical or biologic disturbances in the overlying RPE and outer retina [21].

Zweifel et al. reported on “subretinal drusenoid deposits” in OCT and attempted to correlate imaging findings with postmortem histopathological reports demonstrating the deposition of drusenoid material between the RPE and IS/OS junction [23]. However, no clinical correlation with history of RMD in any imaging modality was provided [23]. In addition, histopathologic studies of eyes affected by AMD have shown degenerative changes in the choriocapillaris [28-31]. Angiography and other blood flow studies have also evidenced an impairment of choroidal circulation [27-30]. Hayreh concluded that the submacular choroid is more vulnerable to chronic ischemia than any other area of the posterior choroid [27]. Further histopathological analysis should include fresh eye samples with a diagnosis of RMD. 

We have previously hypothesized that there is an association between RMD and inflammatory and vascular damage of the choroid [5, 11]. The choroid contains high levels of elements that make up both the alternate and the classic complement pathways, including complement factor H (*CFH*), while in the RPE, low levels of these elements have been identified. The assembly of the membrane attack complex affects the choroid, suggesting that AMD pathogenesis involves local complement activation at the level of the choroid [21, 31]. The activation of the complement system produces an inflammatory process within the choroid, which may lead to atrophic vascular compromise and fibrous replacement and yield the characteristic appearance of RMD in RF, IR, and AF imaging. In addition, this fibrous replacement could explain the impaired filling in FA and ICGA imaging reported in patients with RMD, which is likely secondary to the blockage of fluorescence flow [6, 8, 11, 21]. 

While the pathogenesis is still unclear, there is a reported association between RMD and systemic diseases, such as hypertension and angina, as well as an increased mortality rate independent of comorbidities [11, 12, 19]. In addition, the disproportionate number of females presenting with RMD may suggest a possible autoimmune origin, consistent with the extraordinarily high proportion of women at risk for autoimmune processes [12, 19]. On the other hand, it has also been suggested that there may be an association between AMD and hormonal changes characteristic of menopause, suggesting that low serum levels of estrogen may play an important role in the development of AMD, which led Snow et al. to propose that longer estrogen exposure may be a protective factor against this condition [32]. Thus, if cardiovascular disease and RMD are related, males (who die younger of cardiovascular disease) may be underrepresented in the older population who are identified with the RMD subtype of AMD, explaining the higher incidence of females showing this pattern.

Genetic factors may also play a considerable role in the pathogenesis of RMD, with one study associating the *ARMS2* gene with end-stage AMD as well as RMD [33]. However, the pathogenesis and function of the *ARMS2* allele remains unknown. Exploration of the genotype/phenotype correlation between *ARMS2* and RMD may clarify the significance of the allele and its role in disease. Further speculation must await more data on the function of the *ARMS2* gene and its specific role in AMD [20]. 

Currently, phase 2/3 trials are underway for MC-1101 (hydralazine, MacuClear, Inc.) to treat dry AMD [34]. This drug has been shown in animal studies and preclinical trials to increase choroidal blood flow and remove physiologic waste from the RPE, Bruch’s membrane, and photoreceptor cells. Since RMD is a high-risk AMD phenotype, these trials, if successful, could further support our theory that altered choroidal blood flow is mainly responsible for RMD.

## CONCLUSION

Imaging analysis, histopathological reports, and the possible association between RMD and systemic diseases support our hypothesis that blood flow disturbance in the choroid is involved in the pathogenesis of RMD. Future studies should further investigate the association of RMD with systemic and inflammatory diseases, as well as histopathological examinations and imaging analysis of the same study eye, to to better elucidate the origin and pathophysiology of RMD.

## DISCLOSURE

The authors report no conflicts of interest in this work. This study has been supported by unrestricted funds from Research to Prevent Blindness (RTS), an individual investigator research award from the Foundation Fighting Blindness (RTS), and the Kaplen Foundation (SB).
